# Design Concepts, Fabrication and Advanced Characterization Methods of Innovative Piezoelectric Sensors Based on ZnO Nanowires

**DOI:** 10.3390/s141223539

**Published:** 2014-12-08

**Authors:** Rodolfo Araneo, Antonio Rinaldi, Andrea Notargiacomo, Fabiano Bini, Marialilia Pea, Salvatore Celozzi, Franco Marinozzi, Giampiero Lovat

**Affiliations:** 1 Electrical Engineering Division of DIAEE, University of Rome “La Sapienza”, Rome 00184, Italy; E-Mails: salvatore.celozzi@uniromal.it (S.C.); giampiero.lovat@uniromal.it (G.L.); 2 International Research Center for Mathematics & Mechanics of Complex Systems, University of L'Aquila, Cisterna di Latina (LT) 04012, Italy, and with ENEA, Research Center Casaccia, Rome 00123, Italy; 3 Institute for Photonics and Nanotechnology - CNR, Rome 00156, Italy; E-Mail: marialilia.pea@ifn.cnr.it; 4 Mechanical and Aerospace Engineering Department—DIMA, University of Rome “La Sapienza”, Rome 00184, Italy; E-Mails: fabiano.bini@uniromal.it (F.B.); franco.marinozzi@uniromal.it (EM.)

**Keywords:** zinc oxide, nanowire, atomic force microscopy, FIB machining, conductive AFM, power-law design, FEM analysis

## Abstract

Micro- and nano-scale materials and systems based on zinc oxide are expected to explode in their applications in the electronics and photonics, including nano-arrays of addressable optoelectronic devices and sensors, due to their outstanding properties, including semiconductivity and the presence of a direct bandgap, piezoelectricity, pyroelectricity and biocompatibility. Most applications are based on the cooperative and average response of a large number of ZnO micro/nanostructures. However, in order to assess the quality of the materials and their performance, it is fundamental to characterize and then accurately model the specific electrical and piezoelectric properties of single ZnO structures. In this paper, we report on focused ion beam machined high aspect ratio nanowires and their mechanical and electrical (by means of conductive atomic force microscopy) characterization. Then, we investigate the suitability of new power-law design concepts to accurately model the relevant electrical and mechanical size-effects, whose existence has been emphasized in recent reviews.

## 1. Introduction

Micro- and nano-scale structured materials based on zinc oxide (ZnO) are attractive for applications in electronics and photonics, due to their outstanding properties, including semiconductivity, piezoelectricity, pyroelectricity, direct bandgap and biocompatibility [1–3]. Several kinds of device architectures, including micro-arrays of addressable sensors, are based on a large number of synced ZnO nanowires (NWs) and/or nanopillars to cooperatively generate a large enough response signal or power. The great interest stems from the discovery of superior mechanical and electrical properties of ZnO at the nanoscale, a key element that makes its functional properties exploitable for nanotechnology applications. ZnO nanostructures show outstanding features [4–7], e.g., they are largely deformed by tiny input mechanical forces, may have substantially higher piezoelectric coefficients [8,9] than those exhibited by the bulk material and can withstand extreme deformations without breaking [9,10], thus allowing the generation of high piezopotentials.

However, besides their average response, it is important to characterize the specific mechanical, electrical and piezoelectric properties of a few spatially-localized NWs [11] or possibly single ZnO micro/nanostructures in order to assess the quality of the materials and their performance. In this paper, we present a state-of-the-art review of theoretical and experimental contributions together with the latest results of our ongoing research on ZnO NWs used primarily as sensors.

It is known that the mechanical and piezo-electrical properties of volume-confined materials at the nanoscale are very different from those of conventional bulk samples. For the specific case of ZnO, the bulk material exhibits a brittle behavior with maximum deformations well below 1%, which hinders applications. On the contrary, ZnO nanostructures acquire a ductile behavior and are capable of withstanding large elastic deformations up to 15% without breaking. In addition, besides such a strengthening size effect (SE), ZnO nanostructures also experience a marked stiffening SE for smaller sizes. Furthermore, the piezoelectric coefficients of nanostructures are greatly enhanced by the surface reconstruction of the surface atoms and by high surface-to-volume ratios [9,12]. Based on that evidence, bulk ZnO and nanoscale ZnO can easily be regarded as two totally different materials from a mechanical engineering perspective [8,13,14].

In this framework, the correct characterization of NWs becomes of paramount importance. A route towards the electrical characterization of single isolated ZnO nanostructures is the use of scanning probe-based electrical characterization tools, which make use of metalized nanoscale tips for creating an electrical contact with nanostructures. In order to employ such a technique in the most effective way, several items have to be considered, including:

- the use of proper metal layers, which allow for the formation of ohmic contacts;
- the use of proper masking and/or sacrificial layers during processing (in particular, in the case of focused ion beam milling), with specific concern for their compatibility with the ZnO and with conductive-atomic force microscopy (C-AFM) investigation;
- modifications of the materials induced by processing or characterization.

Finally, the correct modeling of the behavior of NW is fundamental, too. In fact, physically-based simulations offer unique insight into the device behavior by allowing the observation of phenomena that cannot be measured yet on real devices and provide several major advantages: they are predictive, provide useful insights and capture theoretical knowledge in a way that makes this knowledge easily available. Hence, there is a need to develop accurate approaches aimed at fully understanding the coupling effects among mechanical, electrical and piezoelectric properties in nanomaterials and, consequently, to develop novel and more reliable devices.

The paper is structured as follows. In Section 2, we report on the mechanical characterization of NWs introducing a new “double power-law scaling” framework that we recently applied to ZnO NWs [15]. In Section 3, we report on the solutions that allow for reliable C-AFM electrical characterization of ZnO micro-pillars fabricated using the focused ion beam (FIB) technique. Finally, in Section 4, we present an accurate numerical modeling of NWs, based on the classical approach and the finite element method (FEM), which we have applied successfully to several configurations before [15–23]. Our results can provide guidelines for designing high performance piezo-nano-devices for sensors in multiple areas, e.g., electrical, mechanical and chemical processes, paving the way for new strategies for design.

## 2. Mechanical Characterization and Reliability of ZnO NWs

The features, in terms of either piezopotential and the durability of ZnO NWs piezo-devices, largely depend on the extent of the elastic strain that can be withstood by the material without breaking, thus making the extraordinary mechanical properties of ZnO NWs a true “enabling factor” of this nanotechnology. In the past decade, researchers have typically focused primarily on the determination of elastic (*i.e*., the Young's modulus, *E*) and failure (e.g., fracture, fatigue, buckling, *etc*.) properties of ZnO nanostructures for nanogenerators. Substantial evidence has been gathered to confirm the presence of mechanical size effects in ZnO nanowires [8], unmistakably showing the presence of strengthening effects, as well as stiffening effects associated with a size reduction in ZnO NW.

In bulk form, ZnO has a crystalline wurtzite hexagonal structure with a modulus *E* ∼ 140 GPa in the [0001] direction and marked brittle fracture behavior with strains smaller than 1%. In comparison, in spite of some uncertainties and disagreement among published reports, nano-sized samples, primarily NWs and nanobelts (NBs), exhibit substantial differences [1–23], partly because they can be fabricated defect-free (or with a very small amount of defects), as verified via transmission electron microscopy (TEM), scanning electron macroscopy (SEM) and selected-area electron diffraction (SAD) [24–27], and partly because the size effects have been observed and confirmed by several independent studies, both in elastic and fracture/plastic strength properties.

Performing these measurements on 1D nanostructures is a non-trivial task and, due to the lack of standard protocols and equipment, a number of approaches have been applied. It is worthy noting that, following a general trend in nanomechanics [28], the most recent experimental set-up allows *in situ* tests of individual nanoscale samples inside an electron microscope (either TEM-monitored or SEM-monitored) for more accurate positioning of the specimen (relative to fixtures and moving probes) and collection of displacement data in real time during tests. In this section, we present an overview of the main experimental techniques and results, and we address some theoretical aspects that underlie experimental trends and are relevant in a modeling and design perspective.

### 2.1. Overview of Experimental Work

**Figure 1.**
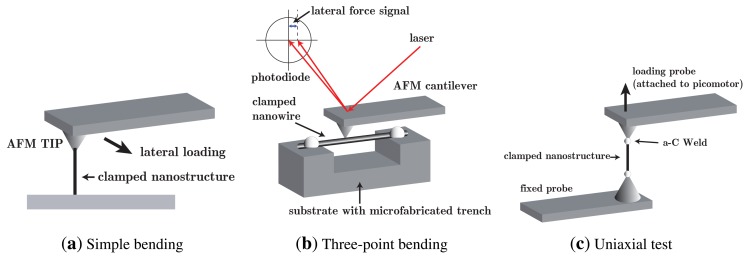
Three experimental configurations used to load nanostructures with an AFM cantilever: (a) simple bending by loading it laterally along the span through an AFM probe driven in lateral force mode; (b) specimen in fixed-fixed conditions subject to three-point bending (either laying flat on a substrate and loaded by the AFM tip laterally, or suspended over a known trench and loaded normally by an AFM tip in contact force mode); (c) specimen in fixed-fixed conditions for uniaxial tests, either tensile or compression (buckling).

A short list and description of methods described in the literature is presented below and illustrated in Figure 1. Early tests were done using resonators [29,30], an *in situ* dynamic technique from the 1990s, where a nanostructure is singly clamped and actuated cyclically inside a TEM (either thermally or electrostatically) at a tunable frequency to achieve mechanical resonance in correspondence to natural vibration frequencies and to estimate elastic properties correspondingly.

AFM-based methods [10,27,31,32] were then introduced since they could be monitored or not inside SEM or TEM, offering unprecedented force and displacement resolutions under static loading. There are three usual experimental configurations to load nanostructures with an AFM cantilever, namely:

(i) specimen laying flat on a substrate in fixed-fixed conditions (Figure 1a) and loaded by the AFM tip in lateral force mode (three-point bending);
(ii) a specimen suspended over a known trench ((Figure 1b) and, fixed at both ends, loaded normally by an AFM tip in contact force mode (three-point bending);
(iii) specimen grown vertically from the substrate (Figure 1c) creating a singly-clamped cantilever loaded along the span or at the tip by the AFM probe driven in lateral force mode (simple bending).

Later on, (instrumented) nanoindentation with a scanning probe capability (e.g., Hysitron Triboscope or alike [33]) was introduced, closely resembling the AFM-based method, but capable of applying larger compressive forces by a massive diamond tip (e.g., Berkovich, conical, cube-corner or other), thus overcoming many limitations associated with AFM cantilevers.

Micromanipulators [34], e.g., a piezoelectric stick-slip robot arm (MMM3A Kleindiek Nanotechnik [34]), were also used as special “fingers” to apply forces on a specimen (like AFM probes) for positioning and displacement measurements inside TEM or SEM. None of these methods, however, could provide tensile data, like a standard, uniform static tensile test. To address this shortcoming, micromechanical systems (MEMS) were proposed for material testing [30,35,36], to perform static tensile tests on a nanoscale specimen suspended between two fixtures fixed at both ends, actuated either thermally or electrostatically.

None of these techniques represents an absolute best, nor can be considered an easy option, because each one requires a rather involved procedure and substantial expertise with the set-up. The most evident proof of that is offered by the inconsistencies that presently exist in published data from different groups. For example, experimentally measured values for Young's modulus, *E*, range from 20 GPa to 250 GPa. A similar scenario holds for fracture properties. A brief author-by-author overview from seven selected research groups is given below.

**Figure 2.**
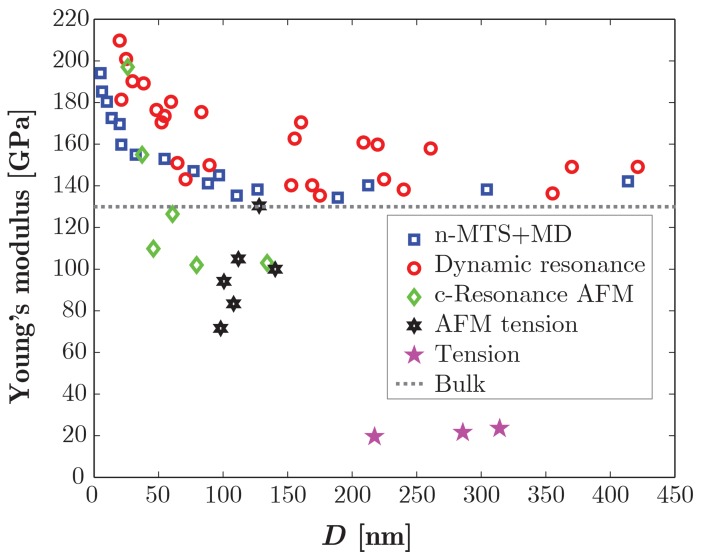
Data plot of Young's modulus from many authors (data from [8]).

1. Espinosa *et al.* [8,9,35,36] developed a TEM-monitored MEMS-based nanoscale material testing stage to perform tensile tests, finding that *E* increased with sample size from 140 GPa to 160 GPa as the nanowire diameter decreased from 80 nm to 20 nm, while it became size-independent and equal to the bulk value 140 GPa for larger wires. The same experiments, instead, indicated size-independent fracture behavior, with measured strengths up to 9.53 GPa and strains up to 6.2% for NW diameters of ∼20–512 nm (Case #1 in Figure 2).
2. Chen *et al.* [37] performed *in situ* dynamic resonance tests like [29], but found values for the elastic modulus of [0001] oriented ZnO NW much larger than those previously reported, together with a marked size-effect in *E* that increased from 140 GPa to 220 GPa as the NW diameter reduced from 550 nm to 17 nm. They also performed bending tests on vertically-grown NWs [38], reporting a size-independent fracture behavior, with strains in the range of 4%–7% and an estimated strength of about 8–12 GPa (Case #2 in Figure 2).
3. Xu *et al.* [10] performed *in situ* SEM-monitored AFM-based tension and buckling tests on single [0001] ZnO NWs. They found that both the tensile modulus (from tension) and bending modulus (from buckling) increased from bulk values to 170 GPa and 200 GPa, respectively, as the NW diameter decreased from 80 to 20 nm. Since the bending modulus seems to increase more rapidly than the tensile modulus, the observed elasticity size effects in ZnO NWs were mainly attributed to surface stiffening. Fracture strength was also found to be size dependent and increased from 4 GPa to 10 GPa at a smaller diameter (Case #3 in Figure 2).
4. Hoffman *et al.* [34] pursued AFM-based bending and tensile tests on ZnO NW in the diameter range of 60–310 nm, finding a size-independent modulus of 80–120 GPa (in disagreement with Chen *et al.* [37], but still within 30% of bulk ZnO) and fracture strains ∼4%–6.5 %, in agreement with Chen *et al.* [38] and much larger than bulk ZnO (Case #4 in Figure 2).
5. Desai and Haque [30] used a MEMS device to characterize the modulus and fracture strength for the quasi-static tensile test and interestingly found a size-independent modulus (*E* ∼ 21 GPa, much less than the bulk) as opposed to a marked size effect in the fracture strain, raising in a near-linear fashion from 5% to 15% when the NW diameter decreased from ∼500 nm to ∼220 nm (Case #5 in Figure 2).
6. Wang and co-workers performed AFM-based (simple) bending tests on single clamped 1D nanostructures and found Young's modulus values of ∼29 GPa for NWs with average diameter of 45 nm [32] and of 55 and 108 GPa for NBs with a width-to-thickness ratio of 2.2 and 1.3, respectively [39]. Previously, they had measured an average E ∼52 GPa for NBs, without observing a size-independent bending [29] from *in situ* dynamic resonance tests. All of these values are much lower than the bulk reference (“bulk” in Figure 2).
7. Ni and Li [27] examined ZnO NBs of a size of 50–140 nm in thickness and 270–700 nm in width, finding E ∼38 GPa from (three-point) bending AFM-based tests, and ∼31 GPa from nano-indentation (the latter case being the sole account of nanoscale plastic deformation in ZnO, albeit limited); they found no size effects and values lower than the bulk indentation.

Although there are other reports available [40,41], the highlighted results are sufficient for the purpose of portraying a “heterogeneous” state-of-the-art in the mechanical characterization of ZnO nanostructures. There are evident conflicts that are expected to be resolved and that require better metrological foundations and improved experimental configurations. Remarkably, only in a few cases [27,34–36], the reported elastic modulus and the fracture strain were measured using the same methodology, while most often, the same research group employed entirely different methods to characterize elastic and fracture properties, thus increasing the probabilities of introducing other critical factors.

Apart from inconsistencies in numerical values, there is also an ambiguity about the size dependence; some of the mentioned studies report a size dependence in ZnO NWs with diameters smaller than a threshold (150 nm or 80 nm), while others do not report any specific size dependence. In general, it is safe to conclude that size effects exist below ∼ 100 nm and that are advantageous to nanogenerators, since reported fracture strains for NWs [10,27,34–38] are about five-times larger those for ZnO in bulk or thin film form, with proportionally larger induced piezoelectric voltages. To explain the elastic effects, for example, Chen *et al.* [37] formulated a core-shell model (based on fitting the experimental data) where the NW is treated as a composite with a shell of higher modulus and a core structure with the bulk Young's modulus. Based on the same idea, Espinosa *et al.* [36] performed molecular dynamics (MD) simulations and predicted a phase transformation in ZnO from the wurtzite phase to a body-centered tetragonal phase at ∼6% strain, which seems in agreement with Wang *et al*. However, [42] never observed this experimentally, allegedly because of testing instabilities (such a transition should be associated with a near-complete load drop, which may not be accommodated in current experimental protocols [36]). The same group also reported TEM evidence of the crucial influence of the atomic surface roughness on the strain localization of fractures in defect-free NWs, which is relevant to the size-effect debate.

**Figure 3.**
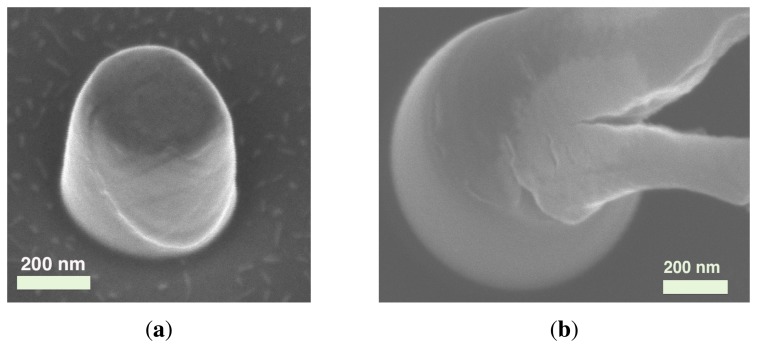
SEM micrograph of compressed ZnO nanopillar. (a) Ductile behavior under a controlled micro-compression test; (b) ductile behavior of a 450-nm diameter (at the root) ZnO NW subject to extreme deformation. The multisite cracking indicates a damage-tolerant behavior.

Micro-compression tests on micropillars are a mainstream choice in nanomechanics [28,33,43–47], which have not received much attention for ZnO, apart from [48]. Instead, the mechanical and piezoelectric behavior of ZnO nanostructures in compression is very relevant, especially in the context of flexible nanogenerators based on lateral NW arrays [6]. According to ongoing work by these authors, the compression of sub-micrometer pillars clearly shows the ductile nature of nanoscale ZnO NWs failing by a single slip, as shown in Figure 3. This technique is also potentially suitable to perform shear [45] and fracture tests on ZnO, which appear to be yet unattempted.

Besides intrinsic properties of ZnO nanostructures, the characterization of fixtures and junctions between NWs and interconnects is very important for reliability, but has not been investigated thoroughly In the cited experimental tests, the free ends of NWs and NBs were fixed by FIB-deposited platinum or via carbonaceous deposits at both ends, but their stiffness and strength remain unexplored or controversial. In fact, while some groups [10,27,34] claim that a thin carbon layer is sufficient to make a firm clamp, others [35,36] disagree and report slippage. Just like deposited clamps, also heterogeneous junctions between substrate and vertical free-standing NWsF were never examined in detail (even when vertically-aligned straight ZnO NW arrays are grown on an excellent epitaxial growth template, such as GaN [0001]); there is a minimal lattice mismatch between the two wurtzite reticules of about 1.8% [49]. However, these two types of defective interfaces are a host to internal stresses and contribute to uncertainties in boundary and loading conditions, causing extrinsic effects that can heavily bias the nanomechanical behavior [43,45]. More importantly, while the static resilience of defect-free ZnO NWs seems outstanding for nanogenerator applications, the junctions/fixtures may represent the weakest link in the mechanical assembly, hindering long-term durability [50]. In this respect, apart from resonator tests aimed at elastic properties, there are only a few reports on the long-term dynamic and fatigue tests. On the other hand, Wang's group explored the fatigue behavior of ceramic ZnO NWs using an *in situ* TEM-monitored resonator, finding no evidence of failure or defect generations after 35 billion cycles. More work is needed to assess the effects at different cycle amplitudes (increasing the stress on the fixtures and discontinuous junctions). Static fracture damage tests on junctions and fixtures may be conducted via instrumented nanoindentation following the *in situ* technique adopted in [45].

Finally, this overview focuses on purely mechanical properties, but piezoelectric characteristics of ZnO NWs and NBs under different loading conditions are also needed and required to track continuously the current-voltage (I-V) characteristics of the system during the mechanical process. This may be achieved by C-AFM, discussed later.

### 2.2. Mechanical Modeling and Design Framework

**Figure 4.**
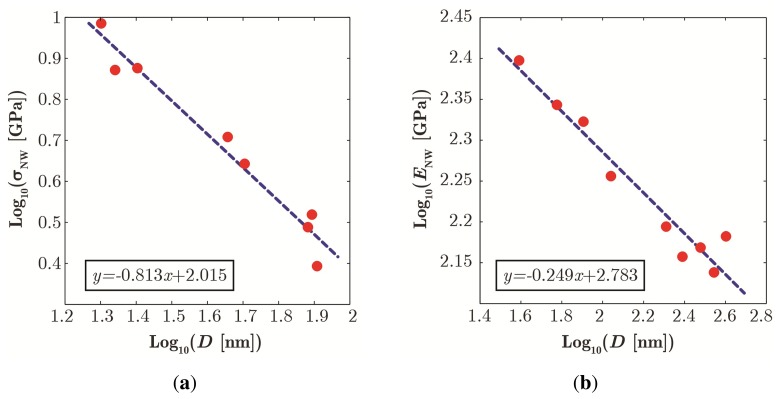
The agreement between experimental data [8] and power-law fit for failure data (a) and stiffness data (b) *versus* NW diameter (*β* = 0.813 and *λ* = 0.249). Plot in log-log scale.

From a mechanical engineering point of view, the presence of size effects on both the elastic (*i.e.*, Young's modulus) and failure (e.g., fracture, fatigue, buckling, *etc.*) properties of ZnO NWs as a function of size *D* is a relevant fact and implies a paradigm shift in the design approach. Since the use of bulk properties as in classical solid mechanics (e.g., Young's modulus and strength assumed to be constant and size-independent) would cause an error, recently, Rinaldi and co-workers [15] proposed that both mechanical size effects can be effectively modeled with power-laws, as in Figure 4. That ushers in the transition from the classical size-independent view to a size-dependent model:

(1) $$\begin{array}{ccc} \begin{array}{c} \text{Classical view}\\ \sigma_{y}=\text{constant}\\ E=\text{constant} \end{array} & \Rightarrow & \begin{array}{c} \text{Power law for size dependence}\\ \sigma_{y}≃D^{-\beta }\\ E≃D^{-\gamma } \end{array} \end{array}$$

While the origins of the power-law are not yet fully understood and continue to attract research interest, the theoretical understanding is progressing. For fracture/strength data, the power-law model has been used and widely accepted for many ductile and brittle nanomaterials, as reflected by the usual tenet “the smaller, the harder” [28,44,51]. Strengthening trends of this kind have been observed not only in single crystal NWs, but also in other nanomaterials [46,47]. This type of power-law trend seems compatible with a generalized Weibull-like framework [28], where higher strength stems from the decreased probability of sampling large defects than the bulk when the NW diameter *D* reduces.

For stiffness data, the theory is less established. Several groups have proposed models based on the Miller–Shenoy hypothesis and other core-shell types of mechanisms tracing back to the presence of surface-induced confinement pressure [28].

At present, regardless of ongoing discussion, albeit empirical, adopting a unified power-law modeling approach for both mechanical size effects in ZnO seems a very attractive and convenient way to account for size effects in the design of sensors and devices based on ZnO nanowires.

## 3. Fabrication and Characterization

In this section, we report on the FIB-assisted fabrication and C-AFM electrical characterization of single ZnO pillars. We used a ZnO film deposited by sputtering, a technique that intrinsically produces a certain amount of defects in the material, which grows with a columnar structure, yet correctly oriented along the direction normal to the wafer substrate, so as to have a fabricated pillar axis parallel to the *c*-axis. Moreover, it is expected that FIB milling induces additional surface defects. Therefore, we point out that the aim of this experimental section is not a straightforward comparison with simulations or theoretical expectations, but rather, the demonstration of the feasibility of the electrical characterization of single isolated ZnO nanostructure fabricated by FIB.

### 3.1. Fabrication

Arrays or single ZnO pillars with a cylindrical, conical or truncated-cone shape were obtained by means of FIB using a Ga^+^ liquid metal ion source operated at 30 kV. A Ti/Au bilayer was used as the top metallic stack acting as both contacting metal and masking material for FIB processing. Ti/Au/Ti and Ti/Au/Al stacks were also used, provided that the top metal is totally consumed during processing or it is removed afterwards by proper etching.

**Figure 5.**
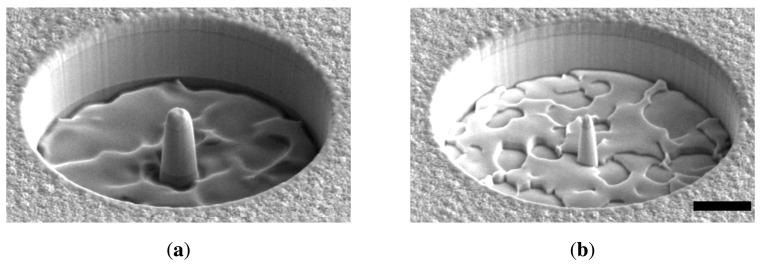
SEM image of ZnO pillars fabricated by focused ion beam on 1800 nm thick sputtered film, using a multilayered metal film as the FIB etching mask. Ga^+^ FIB at 30 keV acceleration energy was used. The scale bar indicates 2 *μ*m. The ZnO pillar in (a) has a 900-nm diameter on top and is 2.2 *μ*m tall, being excavated down into the Si substrate, while the pillar in (b) is 1.7 *μ*m tall and has a diameter on top of 440 nm, resulting in aspect ratios of 2.5 and 3.9, respectively.

Figure 5 shows two isolated pillars, obtained on an almost 1800 nm thick ZnO film deposited by the radio-frequency sputtering technique at room temperature. The deposited material using this technique is typically n-type ZnO. Single pillars located inside circular trenches were fabricated using a “donut” shaped pattern and small ion beam currents (in the range of tens to hundreds of pA), which ensure good control over the shape and surface roughness. A rather thick ohmic metal layer with residual gold on top is present at the pillar apex.

The resulting aspect ratio (AR) is strictly related to the beam size (*i.e.*, the beam current), as well as to the thickness of the masking material and to its masking ability, so it is possible to tune the processing parameters and materials in order to increase both the AR and the control over the pillar shape.

### 3.2. Material Selection for C-AFM

In order to perform reliable C-AFM investigations on ZnO micro/nano-structures, several considerations on the materials are required. Typical coatings used for AFM conductive probes are thin films of noble metals, like gold and platinum, which do not oxidize, allowing “clean” nanoscale semiconductor-metal junctions. However, in the case of ZnO, Au and Pt form rectifying (Schottky) electrical contacts, which hardly allows for the precise measurement of the resistivity of the material. The use of proper metal layers embedded in the ZnO structures is thus required to allow the formation of ohmic contacts.

As reported in Table 1, among the most common metals used in microfabrication, Ti and Al are good candidates as ohmic metals for ZnO [11]. In addition, they are also good masking materials for FIB milling, allowing for the fabrication of high aspect ratio structures. However, these metals display a native oxide layer, which is thick enough to produce an effective barrier to electrical conduction in C-AFM measurements. Moreover, for using FIB processing, it is required that the ohmic metals are deposited on the semiconducting surface before any fabrication steps to avoid surface damage or modifications, which can prevent further processing or affect the electrical characterization. For example, FIB milling produces an amorphous layer in the order of 10–30 nm thick due to ion implantation, and such a layer is capable of passivating the Ti surface, making it resistant to fluorinated gases by reactive ion etching [52]. Furthermore, we have found that the Ga^+^ implanted amorphous layer increases the resistance between the PtIr-coated AFM tip and the metal film greatly. Finally, Ti and Cr are subjected to scanning probe-induced anodization, which occurs at a large negative tip-sample voltage, thus preventing a proper electrical characterization using C-AFM in a wide range of applied biases.

All of these issues point to the use of a proper capping metal layer. Gold can be the material of choice, since it allows for a very low tip-sample resistance in C-AFM measurements, even after FIB processing. Unfortunately, gold has one of the worst FIB masking abilities. Therefore, stacks of several different metals have to be investigated in order to ensure both reliable electrical measurements and fabrication of high aspect ratio structures. A thick-enough Ti/Au bilayer is still adequate to fabricate and characterize micro-/nano-scale pillars of ZnO, as will be shown in the following.

### 3.3. C-AFM Characterization

**Figure 6.**
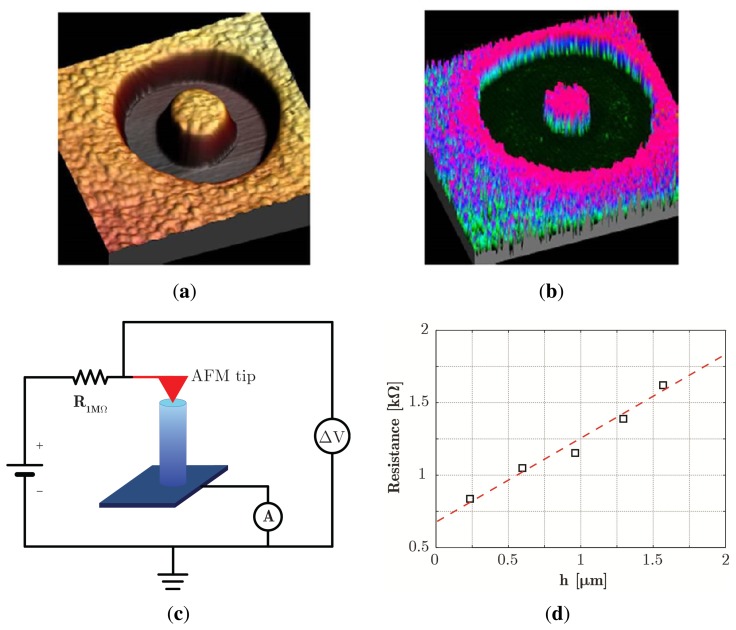
3D view of AFM topography (a) and corresponding 3D view of C-AFM current data map (b) of an FIB-fabricated ZnO pillar with a Ti/Au metal contact on top. The pillar diameter and height are 2 *μ*m and 500 nm, respectively. The C-AFM map has been obtained at a 50-mV bias. The C-AFM measurement set up is shown in (c), where the red arrow indicates the AFM conductive tip; (d) ZnO pillar resistance data measured for pillars with the same diameter (2 *μ*m) and different heights reported *vs.* the pillar height. The red dashed line is the linear fit of the data.

Single ZnO pillars of different heights with a Ti/Au metal contact on top were investigated by C-AFM. A typical AFM 3D image of a pillar is shown in Figure 6a. The corresponding current map, obtained at constant applied bias of 50 mV through a 1 MΩ resistor, is shown in Figure 6d. The current magnitude as measured on top of the pillar is about 45 nA and is almost uniform, indicating that a good tip-sample contact is obtained when the surface sample is covered by gold metal. As a matter of fact, the excavated trench results in being not conductive. In addition, the gold layer is consumed at the borders of the pillar top face, where the underlying non-conducting FIB-implanted Ti layer is exposed; this is consistent with the observation of a smaller pillar diameter in the current map than in the morphological AFM image.

The four-probe resistance of ZnO pillars with the same diameter and different heights was measured by collecting I-V curves using C-AFM. The I-V characteristics measured in a four-probe configuration (the electrical setup is shown in Figure 6c) have a linear behavior, and the corresponding resistance includes the pillar resistance, as well as a contribution of parasitic series resistances due to the tip-sample contact and cables and instrument connections, which can easily be extracted from the data trends. It is worth noting that the conductance of the material used includes average effects due to structural or fabrication-induced defects greatly influencing the measured resistance values, which may differ significantly from that of an ideal single crystal material.

The linear dependence of the resistance on the pillar height (as shown in Figure 6d), which is expected for an ohmic conductor, demonstrates the feasibility of a reliable electrical characterization by C-AFM measurement of ZnO nanostructured materials by FIB nano-machining.

## 4. Numerical Model

The electro-mechanical behavior of the piezoelectric semiconductor NW can be conveniently described through the consistent physical mathematical model offered by the classical approach that has been successfully applied to many devices [4,17,18,23,53,54]. This approach allows one to derive a system of fully-coupled non-linear partial differential equations that describe the mechanical deformation of the wire, as well as the charge transport inside the NW. The mechanical elastic behavior is governed by Newton's law, while the electric voltage behavior obeys Poisson's equation [55–57], respectively:

(2a) $$\nabla \cdot ([c^{\text{E}}]\varepsilon )=-f_{\upsilon ,\text{piezo}}$$

(2b) $$\nabla \cdot (\kappa_{0}[\kappa^{\varepsilon }]E)=q(N_{\text{d}}^{+}+p-n)+\rho_{\text{piezo}},$$

with *ε* the strain tensor, **E** the electric field vector, *n* and *p* the free electron and hole concentrations, [**c**^E^] the mechanical stiffness matrix, [***κ****^ε^*] the relative permittivity matrix and $N_{\text{d}}^{+}$ the ionized positive donors (*q* indicates the absolute value of the electronic charge).

The two forcing terms in Equations (2) are due to the direct and inverse piezoelectric effect, which is accounted for by the piezoelectric coefficients matrix [**e**]. In fact, the body forces per unit volume are generated by the inverse piezoelectric effect, being **f***_v_* = − ∇ · ([**e**]^T^ **E**), and the volumetric charges are produced by the direct piezoelectric effect, with *ρ*_piezo_ = − ∇ · ([**e**] *ε*).

Under compressive or tensile strain, mobile electrons redistribute under thermodynamic equilibrium according to the Fermi-Dirac statistics (which can be approximated by the Boltzmann distribution in the case of non-degenerate semiconductors), and consequently, the conduction band level is a function of space coordinates **r** [17–21,53,54]. The band edge shift, which is equal to $\Delta E(r)=-qV+a_{\text{def}}\frac{\Delta \upsilon }{\upsilon_{0}}$, is the sum of the electrostatic energy term *qV* and the deformation potential term *a_def_* Δ*υ/υ*_0_, where *V* is the electrostatic potential, Δ*υ/υ*_0_ is the relative volume change and *a*_def_ is the deformation potential constant [54]. In addition, also the activation process of the donors and acceptors is modified by the deformation of the conduction and valence band edge [17,18,53,54,58].

The coupled nonlinear set of Equations (2) has been solved by means of the finite element method (FEM) using the Newton nonlinear iteration method [59]. For consistency with previous analyses, we consider a total-bottom contact [17–21,53,54], because NWs are typically grown on seed layers whose electrical conductance is generally rather high, as previously explained. The simulation domain consists of the NW, where the coupled piezoelectric model has been solved, surrounded by free space, where the simple Laplace equation has been enforced (modeled as an infinite nonsolid medium).

The values of the ZnO parameters used in our calculations are as follows. Since a transversely isotropic approximation applies to the hexagonal crystal of ZnO wurtzite, so that the behavior is isotropic on the basal plane, the elastic stiffness tensor *c_ijkl_* is here expressed according to the Voigt notation as a 6 × 6 matrix, where only five independent elastic constants are present, *i.e.*, $c_{11}^{\text{E}}=c_{22}^{\text{E}}$, $c_{33}^{\text{E}}$, $c_{12}^{\text{E}}$, $c_{13}^{\text{E}}=c_{23}^{\text{E}}$ and $c_{44}^{\text{E}}=c_{55}^{\text{E}}$, being $c_{66}^{\text{E}}=\left(c_{11}^{\text{E}}-c_{22}^{\text{E}}\right)/2$. The elastic constants $c_{11}^{\text{E}}$ and $c_{33}^{\text{E}}$ refer to the [1000] and [0001] crystallographic directions, *i.e.*, the *a* and *b* basal axes and the *c*-axis of ZnO, respectively.

The anisotropy between the basal plane and oaxis in bulk ZnO is often deemed negligible for practical purposes [3], and a fully isotropic approximation has been almost universally adopted in piezotronics, delivering the following expressions between the elasticity coefficients and the more common Young's modulus and Poisson ratio:

(3a) $$c_{11}^{\text{E}}=c_{22}^{\text{E}}=c_{33}^{\text{E}}=E_{\text{BULK}}(1-\nu_{\text{BULK}}^{2})\cdot Y=206.135[\text{GPa}]$$

(3b) $$c_{12}^{\text{E}}=c_{13}^{\text{E}}=c_{23}^{\text{E}}=E_{\text{BULK}}(\nu_{\text{BULK}}+\nu_{\text{BULK}}^{2})\cdot Y=110.509[\text{GPa}]$$

(3c) $$c_{44}^{\text{E}}=c_{55}^{\text{E}}=c_{66}^{\text{E}}=\frac{E_{\text{BULK}}}{2(1+\nu_{\text{BULK}})}=47.813[\text{GPa}],$$

with the bulk Young's modulus *E*_BULK_ = 129 GPa, the Poisson ratio *v*_BULK_ = 0.35 and $Y={\left(1-3\nu_{\text{BULK}}^{2}-2\nu_{\text{BULK}}^{3}\right)}^{-1}$ .

These equations provide the terms of comparison for the refined model of the ZnO NW accounting for the stiffening size-scale effect. In fact, the key of the proposed approach is to insert into Equation (3) a size-dependent Young's modulus *E*(*D*) that depends parametrically on the diameter *D* through the fitted analytical power-law, keeping unchanged the Poisson ratio. Hence, the new expressions are:

(4a) $$c_{11}^{\text{E}}=c_{22}^{\text{E}}=c_{33}^{\text{E}}=E(D)(1-\nu_{\text{BULK}}^{2})\cdot Y$$

(4b) $$c_{12}^{\text{E}}=c_{13}^{\text{E}}=c_{23}^{\text{E}}=E(D)(\nu_{\text{BULK}}+\nu_{\text{BULK}}^{2})\cdot Y$$

(4c) $$c_{44}^{\text{E}}=c_{55}^{\text{E}}=c_{66}^{\text{E}}=\frac{E(D)}{2(1+\nu_{\text{BULK}})}.$$

**Figure 7.**
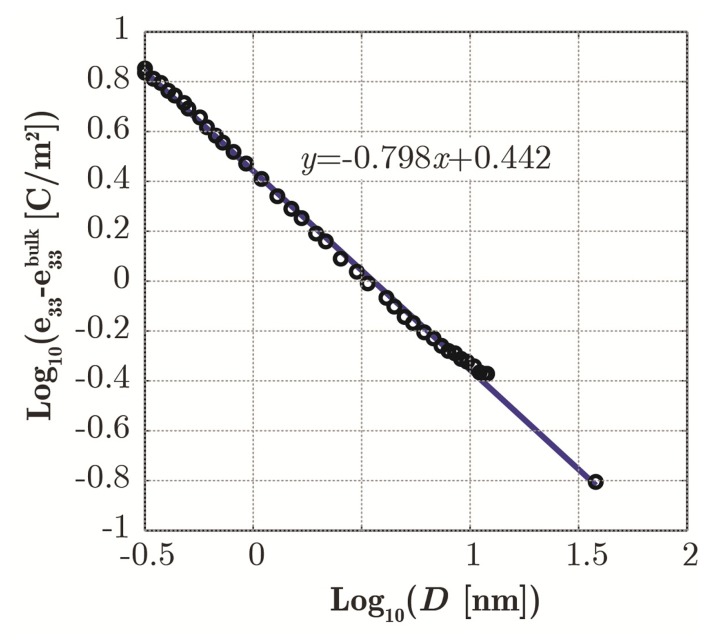
The agreement between data from [9,12] and the power-law fit for piezoelectric constant *e*_33_ *versus* NW diameter (*β* = − 0.798). Plot in log-log scale.

Similar reasoning has been applied with regard to the modeling of the piezoelectric behavior. Usually, the bulk values have been assigned to the piezoelectric coefficients in previous simulations [17–21,53,54]: they are *e*_31_ = *e*_32_ = −0.51 C/m^2^, *e*_33_ = 1.22 C/m^2^, *e*_15_ = *e*_24_ = −0.45 C/m^2^ [60]. On the contrary, starting from experimental and theoretical values [9,12], here, we assume that the *e*_33_ parameter follows a power-law trend as depicted in Figure 7. The other two coefficients, less important under uniaxial compressive/tensile strain, are assumed to be fixed and equal to the bulk values.

The relative dielectric permittivity tensor is considered uniaxial with only two independent dielectric constants, *i.e.*, $\kappa_{11}^{\varepsilon }=\kappa_{22}^{\varepsilon }=7.77$ and $\kappa_{33}^{\varepsilon }=8.91$ [61], since refractive-index analyses have shown only two different refractive indices for polarization parallel (ordinary) and perpendicular (extraordinary) to the *c*-axis.

As concerns the remaining parameters required by the model, the effective electron mass is *m*_e_ = 0.28*m*_0_ [62], with *m*_0_ the mass of free electrons; the deformation potential constant is *a*_c_ = − 6.05eV [63]; the distance between the donor (acceptor) energy level and conduction (valence) band is Δ*ε*_D_ = Δ*ε*_A_ = 35 meV [61]; the temperature is *T* = 300 K; and the band gap is *ε*_g_ = 3.4eV [64].

### 4.1. Rationale

**Figure 8.**
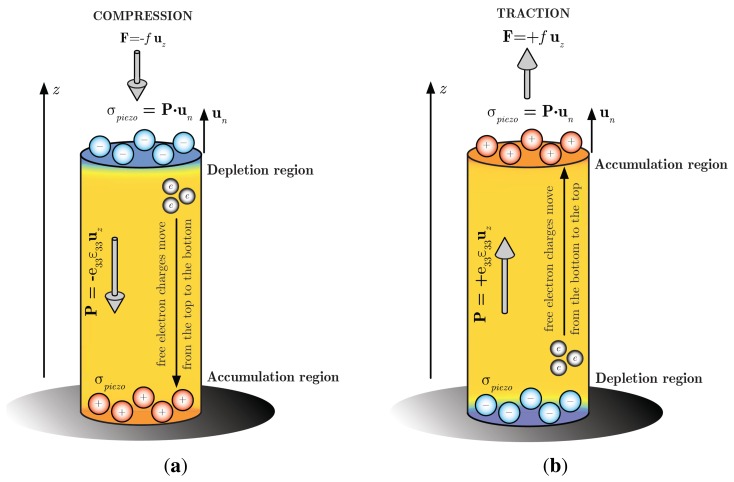
Schematic of the NW under compressive (a) and tensile strain (b).

A simple theoretical explanation of the depletion/accumulation region that is induced at the top of the NW under small compressive/tensile strains (Figure 8) can be obtained from an approximate solution of the aforementioned relevant equations. For small voltages, the volume charge density can be expressed retaining only the first term of the Fourier series as:

(5) $$\rho =q[N_{\text{D}}^{+}-n]=q\left[N_{\text{D}}^{+}-N_{\text{c}}\exp\left(\frac{E_{c}}{k_{\text{B}}T}-\frac{qV}{k_{\text{B}}T}\right)\right]≅-\frac{N_{\text{D}}^{+}q^{2}V}{k_{\text{B}}T}+O[V^{2}],$$

where *E_c_* and *N_c_* are, respectively, the conduction band energy and the number of states in the conduction band, and *k*_B_ is the Boltzmann constant.

**Figure 9.**
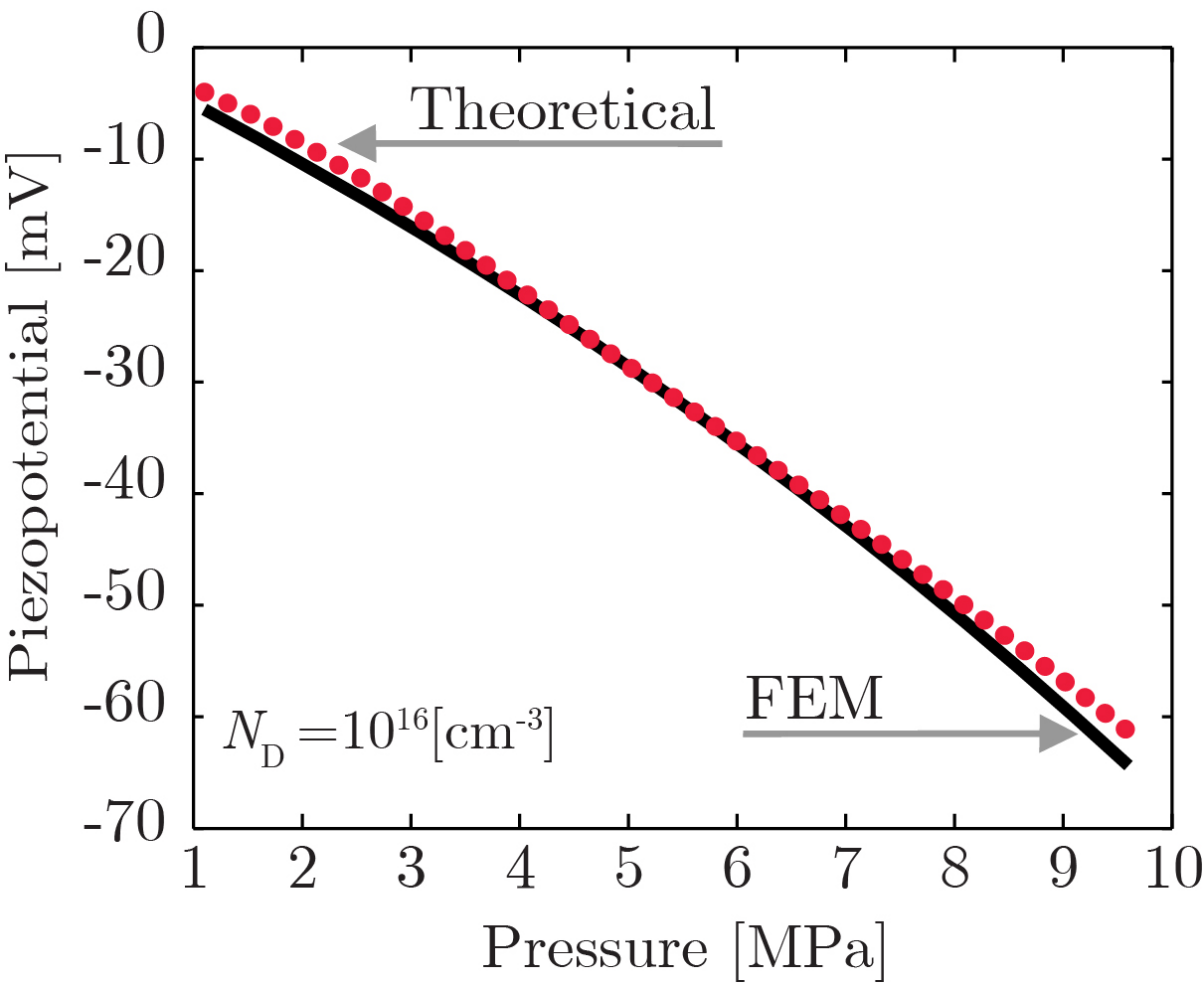
Comparison of the theoretical prediction of the piezopotential at the tip of the NW *versus* the numerical results obtained by means of the FEM model.

Assuming the strain constant for a uniaxial compression $\varepsilon_{33}≅-\frac{F_{\text{load}}}{ES}$ (*i.e.*, neglecting the strain induced by the mechanical fixed constraint at the base of the nanowire), the piezoelectric polarization is assumed constant and equal to *P* ≅ *e*_33_*ε*_33_. The polarization introduces a surface charge density at the top of the NW equal to *σ*_piezo_ = **P** · **n** = ∓ *P*, negative/positive depending if the NW is subject to compressive/tensile strain, as shown in Figure 9. The induced piezoelectric charges make the free negative charges in the conduction band move from the top (bottom) towards the bottom (top) of the NW under compressive (tensile) strain, creating a depletion (accumulation) region at the top of the NW that we assume of depth equal to *δ*, the unknown to be computed. The Poisson equation along the *z*-axis (assuming a 1D problem) reads:

(6) $$-\kappa_{33}\frac{d^{2}V}{dz^{2}}=\rho$$

since we assume that there is no piezoelectric volumetric charge *ρ*_piezo_ = − ∇ · P ≅ 0. The general solution is readily obtained as *V* (*ζ*) = *k*_1_ exp (*αζ*) + *k*_2_ exp (−*αζ*), with *k*_1_ and *k*_2_ arbitrary constants, $\alpha =q\sqrt{\frac{N_{\text{D}}}{k_{\text{b}}T\kappa_{33}}}$, and where *ζ* is the translated abscissa with origin at *z* = *L* − *δ* (at the beginning of the depletion/accumulation region). To obtain the particular solution, we impose the boundary conditions *V* (0) = 0, *i.e.*, an unperturbed conduction band at the bottom of the depletion/accumulation region, and ${D\cdot u_{\text{n}}=\sigma_{\text{piezo}}=\kappa_{33}\frac{dV(z)}{dz}|}_{\zeta =\delta }$ at the top surface of the NW. With some manipulations, we obtain:

(7) $$V(z)=\frac{\sigma_{\text{piezo}}}{\alpha \kappa_{33}}\text{sech}\;(\alpha \delta )\sinh\;(\alpha \zeta )$$

By assuming total electrostatic induction between the polarization charge and the free electron charge, we impose:

(8) $$\int_{0}^{\delta }n\text{dz}=\int_{0}^{\delta }q\left(N_{\text{D}}-\frac{N_{\text{D}}qV}{k_{\text{B}}T}\right)\text{dz}=-\sigma_{\text{piezo}}$$

in order to compute *δ*, which can be obtained by solving numerically the following transcendental equation:

(9) $$qN_{\text{D}}\delta -\sigma_{\text{piezo}}\text{sech}\;(\alpha \delta )=-2\sigma_{\text{piezo}}.$$

Figure 9 shows the comparison between the piezopotential at the tip of the nanowire under a compressive pressure ranging from 1 MPa to 10 MPa, computed theoretically and by means of the numerical FEM model. It can be observed that the theoretical model allows for a simple understanding of the physics at the base of the piezoelectric phenomena with reasonable accuracy in the low-strain assumption.

### 4.2. Simulation Results

Hereafter, we show some interesting results obtained through the aforementioned numerical approach concerning the potential consequences of the size-scale effects (SE). In the field of sensors, the fact that ZnO NWs present strengthening and stiffening trends proportional to the inverse root of *D* has two important implications on the mechanical and electrical performance of NWs: on the one hand, the strengthening effect allows one to achieve much higher strains at low diameters *D* (much higher than those sustainable by the bulk material) and, on the other hand, the stiffening effect systematically induces lower strains and, consequently, reduces the bulk estimates. The scenario is made more complicated by the SE of the piezoelectric constant *e*_33_, which is the dominant parameter among the three piezoelectric coefficients, being the primary one responsible for the electro-mechanical coupling. In particular, the piezoelectric output voltage (called piezopotential), which is proportional to the piezoelectric coefficient through the direct-piezoelectric effect, can be improved (even by one or two orders of magnitude) by reducing the size of NWs. Moreover, there is also a parallel increase of the piezoelectric stiffening due to the increase of the inverse-piezoelectric effect. Hence, it is evident that it is mandatory to find a trade-off among the three SEs that are somehow clashing.

In the following examples, we consider a conical NW (with a base angle equal to 5°), which has been shown to present very interesting features [15,17], with an aspect ratio *k* = *H/D* = 5, where *D* is the base diameter and *H* the height of the NW. The bulk model is compared with two SE models through a parametric study where the applied pressure *p* and the root diameter *D* of a free standing NW loaded at the tip are varied in the range 0.1–2 GPa and 20–300 nm respectively. In the first model (denoted as Model 1), we introduce only the SE on the piezoelectric constant *e*_33_ while, in the second model (denoted as Model 2), we introduce also the SE on the Young's modulus. We underline that the chosen pressure range allows for achieving the 10%–15% maximum strain levels reported experimentally over the selected size range, while remaining within material strength prescription ensured by Equation (1). Since the electrical effects depend also on the NW shape, conical NWs are compared while scaling the NW size in a self-similar manner at the constant aspect ratio *k*, to weigh the mechanical SE against both NW shape and size. Preserving the shape during scaling and limiting *k* is also technologically meaningful to avoid elastic buckling of the NW at higher pressures [65].

The two main outputs quantifying the piezo performance of an NW for a given mechanical load are the (maximum) piezopotential *V* and the efficiency factor *ξ*, where the latter conveys the capacity to produce a piezoelectric effect and is defined as the ratio between the electrostatic energy *W_e_* and the total energy (the sum of *W_e_* and the mechanical strain energy *W_m_*) as:

(10) $$\xi =\frac{W_{e}}{W_{e}+W_{m}}$$

with:

(11a) $$W_{e}=\int_{\text{WHOLE SPACE}}\frac{1}{2}\kappa_{ij}E_{i}E_{j}\;d\Omega$$

(12b) $$W_{m}=\int_{NW}\frac{1}{2}C_{ij}\varepsilon_{i}\varepsilon_{j}\;d\Omega$$

It should be noted that the efficiency *ξ* measures the capacity to produce a piezoelectric effect, and the electrostatic term *W_e_* encompasses both the contribution within the NW and in the surroundings.

**Figure 10.**
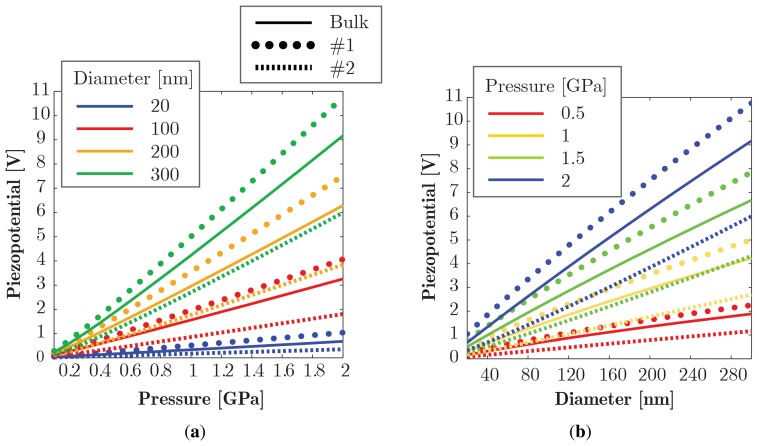
Simulation results comparing the piezopotential *V* from bulk *versus* size effect (SE) models over a *D* range of 20–300 nm and pressure *p* from 0.1 GPa up to 2 GPa, for conical NWs with aspect ratio *k* = 5: (a) piezopotential *versus* pressure; (b) piezopotential *versus* diameter.

Figure 10 shows the simulation results for the piezopotential *V*. The results indicate that, if the piezoelectric SE systematically increases the bulk estimate of output voltage as a consequence of the higher piezoelectric coefficient *e*_33_, with an increase of even 17% at high *p* and *D*, the mechanical SE dramatically reduces the piezopotential as a consequence of the lower strains induced by the stiffening effect, with an overall reduction with respect to the bulk estimate of even 34%. The piezopotential expectedly increases by applying higher pressure, but the existence of the SE counters this increase, reducing the benefit at larger pressure *p* at small scales compared to the bulk.

**Figure 11.**
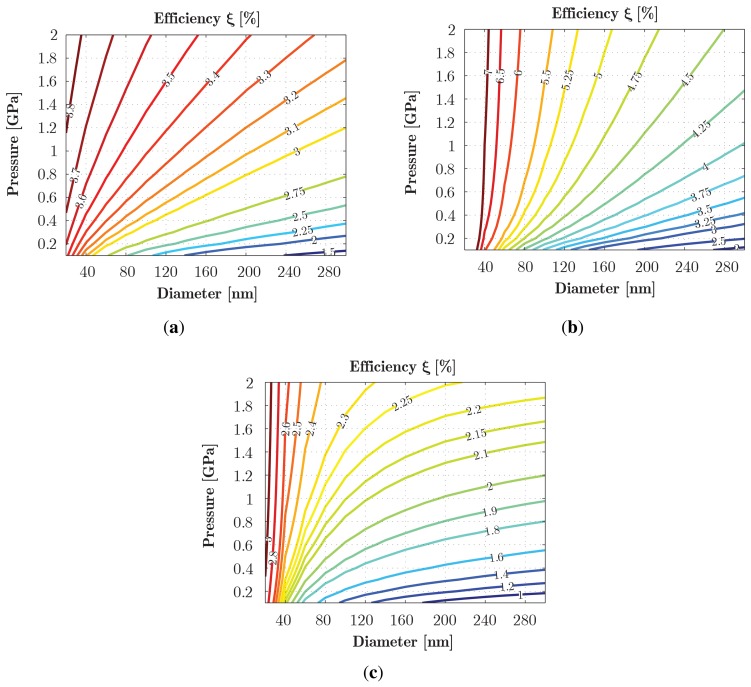
Simulation results comparing the efficiency *ξ* from bulk *versus* SE models over a *D* range of 20-300 nm and pressure *p* from 0.1 GPa up to 2 GPa, for conical NWs with aspect ratio *k* = 5: (a) bulk model; (b) scale-effect on the piezoelectric constant; (c) scale-effect on the piezoelectric constant and the Young's modulus.

This viewpoint is reinforced in consideration of the efficiency results shown in Figure 11, which add greater insight. Furthermore, in this case, if the SE on the piezopotential coefficient boosts dramatically the efficiency that almost doubles with respect to the bulk case, the introduction of the SE on the mechanical properties of the NW leads to a systematic reduction in efficiency that, despite the improvement in the *e*_33_, becomes considerable lower than the bulk estimate. It should be observed that smaller NWs still exhibit an increase in the efficiency reported in prior literature [17], but the SEs strongly modulate the actual benefit. In practice, the choice of NW size in actual sensor devices is a trade-off between mechanical strengthening, piezoelectric enhancement and the required performance.

## 5. Conclusions

We have presented a thorough discussion of the most relevant results of our theoretical and experimental research on ZnO NWs for sensor applications. We have initially performed an overview about the mechanical characterization and reliability of NWs, trying to convey the multidisciplinary nature of this research and the importance of acknowledging both electrical and mechanical size effects. To account simultaneously for such size effects, we have proposed using individual power-law scaling functions, which are convenient to implement and seem to accurately capture the experimental trends. We have also proposed a characterization of ZnO NWs to experimentally extract model parameters based on a reliable C-AFM technique. Finally, we have presented the numerical scheme that we have successfully used for the characterization of several devices.

## Figures and Tables

**Table 1.** FIB milling rates, masking ability and the kind of electrical contact to the n-type ZnO [11] data for a selection of metals commonly used in microelectronics and MEMS technology.

	**Ti**	**Al**	**Cr**	**Au**	**ZnO**
FIB milling rate (Relative to Ti rate set to 100)	100	150	200	275	150
Masking ability	Best	Good	Good	Worst	–
Typical electrical contact with ZnO	Ohmic	Ohmic	Schottky	Schottky	–
